# Solvent influence on the crystal structures of new cadmium tri-*tert*-but­oxy­silane­thiol­ate com­plexes with 1,4-bis­(3-amino­prop­yl)pi­per­a­zine: luminescence and anti­fungal activity

**DOI:** 10.1107/S2053229623005442

**Published:** 2023-07-05

**Authors:** Daria Kowalkowska-Zedler, Piotr Bruździak, Zbigniew Hnatejko, Renata Łyszczek, Anna Brillowska-Dąbrowska, Łukasz Ponikiewski, Bartosz Cieśla, Agnieszka Pladzyk

**Affiliations:** a Department of Inorganic Chemistry, Gdańsk University of Technology, Narutowicza 11/12, Gdańsk 80-233, Poland; b Department of Physical Chemistry, Gdańsk University of Technology, Narutowicza 11/12, Gdańsk 80-233, Poland; c Department of Rare Earths, Adam Mickiewicz University in Poznań, Uniwersytetu Poznańskiego 8, Poznań 61-614, Poland; d Department of General and Coordination Chemistry, Maria Curie-Skłodowska University, PL. M.C. Skłodowska-Curie 2, Lublin 20-031, Poland; e Department of Molecular Biotechnology and Microbiology, Gdańsk University of Technology, Narutowicza 11/12, Gdańsk 80-233, Poland; Wilfrid Laurier University, Waterloo, Ontario, Canada

**Keywords:** anti­fungal activity, luminescence, solvent influence, crystal structure, hydrogen bonding, thiol, piperazine

## Abstract

Monocrystals of dinuclear μ-1,4-bis­(3-amino­prop­yl)pi­per­a­zine-κ^4^ *N* ^1^,*N* ^1′^:*N* ^4^,*N* ^4′^-bis­[bis­(tri-*tert*-but­oxy­silane­thiol­ato-κ*S*)cadmium(II)], [Cd_2_(C_12_H_27_O_3_SSi)_4_(C_10_H_24_N_4_)] or [Cd_2_{SSi(O*t*Bu)_3_}_4_(μ-BAPP)], **1**, and polynuclear *catena*-poly[[bis­(tri-*tert*-but­oxy­silane­thiol­ato-κ*S*)cadmium(II)]-μ-1,4-bis­(3-amino­prop­yl)pi­per­a­zine-κ^2^ *N* ^1′^:*N* ^4′^], [Cd(C_12_H_27_O_3_SSi)_2_(C_10_H_24_N_4_)]_*n*_ or [Cd{SSi(O*t*Bu)_3_}_2_(μ-BAPP)]_*n*_, **2**, with 1,4-bis­(3-amino­prop­yl)pi­per­a­zine (BAPP) and tri-*tert*-but­oxy­silane­thiol­ate ligands, were obtained from the same ratio of reactants, but with different solvents used for the crystallization processes. The structures and properties of both com­plexes were characterized using elemental analysis, X-ray diffraction and FT–IR, ^1^H NMR and luminescence spectroscopy. Applied density functional theory (DFT) com­putational methods and noncovalent inter­action (NCI) analysis were used for geometry optimization and visualization of the inter­actions between the metallic centres and their surroundings. The X-ray analysis revealed four-coordinate Cd^II^ centres bound to two S atoms of the silanethiolate groups and two N atoms of the BAPP ligand; however, it chelates to tertiary and primary N atoms in **1**, whilst in **2** it does not chelate and bonds only to *R*NH_2_. The photoluminescence properties of com­plexes **1** and **2** result from free-ligand emission and differ significantly from each other with respect to emission intensity. Additionally, anti­fungal activity was investigated against 18 isolates of fungi. Compound **1** strongly inhibited the growth of three dermato­phytes: *Epidermophyton floccosum*, *Microsporum canis* and *Trichophyton rubrum*.

## Abstract 2

Two cadmium tri-*tert*-but­oxy­silane­thiol­ates with 1,4-bis­(3-amino­prop­yl)pi­per­a­zine were obtained from identical molar ratios of reagents in different solvents. The structures of the com­plexes have a direct impact on their spectral properties, as well as their anti­fungal properties.

## Introduction

Piperazine is found to be a structural com­ponent of com­pounds that exhibit inter­esting properties. Due to its structural similarity to glucose and cyclo­dextrins, as well as the ability of pi­per­a­zine N atoms to bind with DNA, this mol­ecule and its derivatives have attracted remarkable inter­est as ligands in the synthesis of com­pounds that exhibit anti­cancer (Nemati *et al.*, 2021 ▸; Ragab *et al.*, 2022 ▸), anti­microbial (Niemeyer *et al.*, 1979 ▸; Keypour *et al.*, 2008 ▸) and anti­malarial activity (Ryckebusch *et al.*, 2003 ▸; Guillon *et al.*, 2017 ▸). Piperazine derivatives are also used in the synthesis of metal coordination com­pounds. One of the promising groups of such com­plexes are based on cadmium. Despite the toxic properties of the metal itself, the com­pounds are still studied for the purpose of assessing their biological activity. However, this is not the only area in which the applicability of cadmium com­pounds is under investigation, they have also been assessed for their structural, photo­chemical and catalytic suitability (Wing-Wah Yam *et al.*, 1999 ▸; Singh *et al.*, 2015 ▸; Keypour *et al.*, 2009 ▸; Półrolniczak *et al.*, 2018 ▸; Buta *et al.*, 2021 ▸). 
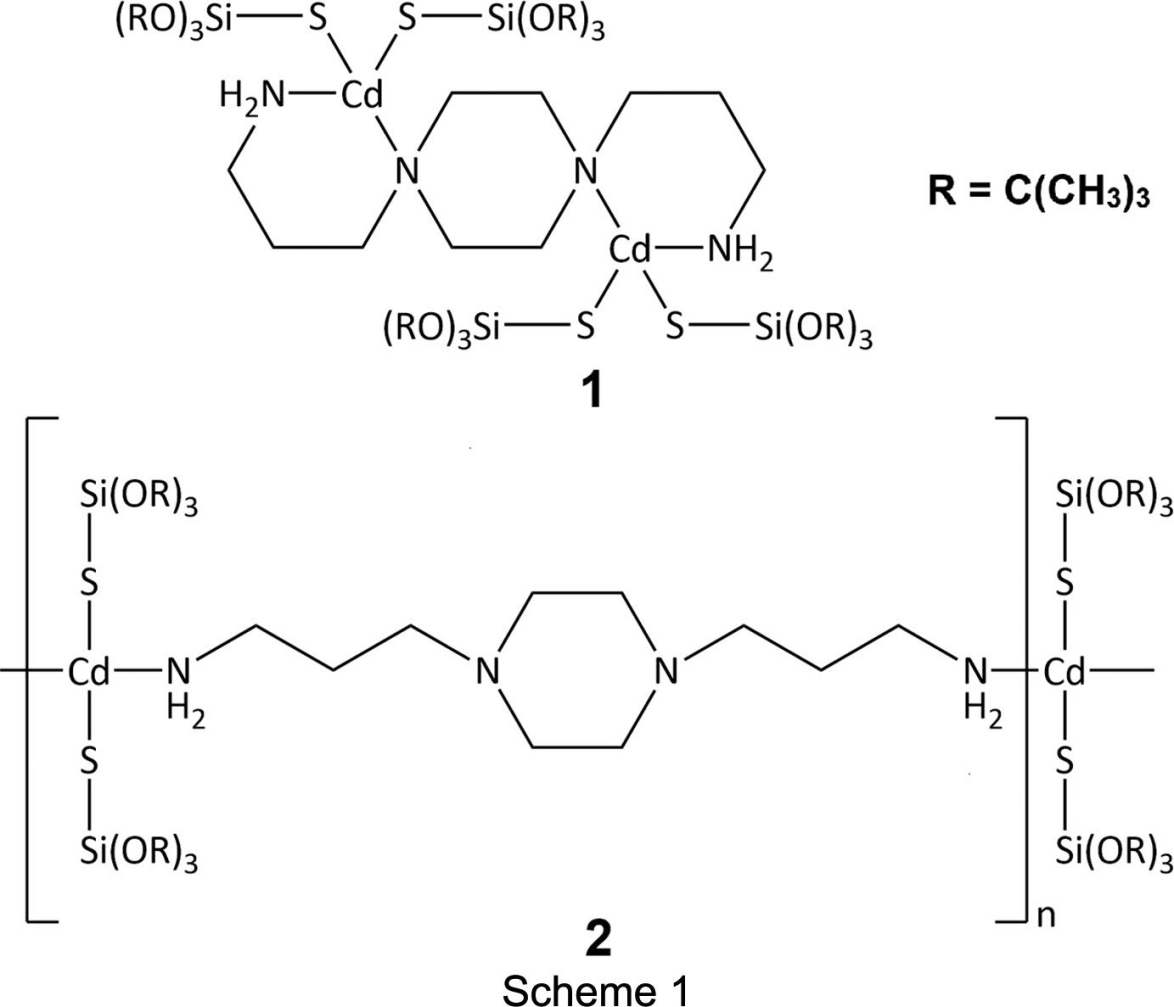


A detailed analysis of the literature has shown that there are no Cd com­plexes that contain pi­per­a­zine derivatives and thiol­ate residues. This fact is somewhat surprising, as numerous examples show that the presence of thiol­ate residues in heteroleptic com­plexes often confers additional physicochemical properties (Henkel *et al.*, 2004 ▸; Resta *et al.*, 2010 ▸; Gennari *et al.*, 2020 ▸; Korkola *et al.*, 2023 ▸). Therefore, we have made attempts to synthesize cadmium com­pounds containing both thiol­ate and pi­per­a­zine derivatives as ligands.

Our scientific group is investigating alk­oxy- and aryl­oxy­sil­ane­thiols, (*R*O)_3_SiSH, which are analogues of organic thiols, *R*SH. They contain a C—O—Si—S—H moiety instead of a C—S bond and act as both *S*-terminal and *O*,*S*-chelating ligands in the synthesis of coordination com­pounds (Pladzyk *et al.*, 2021 ▸). The com­pound commonly used by us in the syntheses of silane­thiol­ates is tri-*tert*-but­oxy­silane­thiol, (*t*BuO)_3_SiSH (TBST). Due to its spatial structure, it shows remarkable resistance towards hydrolysis of the Si—S bonds, allowing for synthesis under atmospheric conditions, giving mono-, di- or polymeric com­plexes of various metals. Our research focuses on the design and preparation of heteroleptic metal silane­thiol­ates that exhibit luminescence, magnetic and/or catalytic features (Pladzyk *et al.*, 2021 ▸). One group of such derivatives is cadmium silane­thiol­ates and our current research is directed towards recognizing the role of 1,4-bis­(3-amino­prop­yl)pi­per­a­zine (BAPP) in the structural, spectral and cytotoxic properties of cadmium tri-*tert*-but­oxy­silane­thiol­ates containing this ligand. For this purpose, we conducted reactions using the same molar ratios of cadmium silane­thiol­ate, [Cd{SSi(O*t*Bu)_3_}_2_]_2_ (Wojnowski *et al.*, 1992 ▸), and BAPP in two different solvent systems. As a result, two new cadmium(II) com­plexes were obtained, namely, [Cd_2_{SSi(O*t*Bu)_3_}_4_(μ-BAPP)], **1**, and [Cd{SSi(O*t*Bu)_3_}_2_(μ-BAPP)]_*n*_, **2**, and their crystal structures, combined with their theoretical studies, spectral characterization and cytotoxic characteristics, are presented below.

## Experimental

### General procedures

The elemental analyses (C, H, S and N contents) were performed with a Vario EL Cube CHNS apparatus. The FT–IR spectra were measured for crystalline com­pounds **1** and **2** in the range 4000–400 cm^−1^ with a Nicolet iS50 FT–IR spectrometer equipped with a Specac Quest single-reflection diamond attenuated total reflectance (ATR) accessory. ^1^H NMR spectra were recorded in solution on a Bruker AV400 MHz spectrometer [external standard: tetra­methyl­silane (TMS)]. Luminescence spectra in the UV–Vis range were recorded at room temperature on a Hitachi F7000 spectrophotometer equipped with a 150 W xenon lamp as the light source. Thermal analysis of both cadmium com­plexes in air was performed by thermogravimetric (TG) and differential scanning calorimetry (DSC) methods using a Setsys 16/18 Setaram analyzer. The samples (about 8 mg) were heated in aluminium crucibles in the temperature range 30–1000 °C in flowing air with a heating rate of 10 °C min^−1^. Thermal analysis under a nitro­gen atmosphere was performed using a Q5000 TA apparatus. Samples of about 20 mg were heated to 700 °C at a rate of 20 °C min^−1^ in flowing nitro­gen.

### Syntheses

[Cd{SSi(O*t*Bu)_3_}_2_]_2_ was obtained according to procedures described previously (Wojnowski *et al.*, 1992 ▸). All other reagents were obtained commercially and were used with no further purification.

#### [Cd_2_{SSi(O*t*Bu)_3_}_4_(μ-BAPP)], 1

A portion of BAPP (0.082 ml, 0.4 mmol) was added to the solution of [Cd{SSi(O*t*Bu)_3_}_2_]_2_ (0.1 mmol, 0.13 g) in methanol (25 ml). The mixture was left at 4 °C for crystallization and after one week colourless crystals of **1** were obtained (yield 60%; m.p. 163.5–164.9 °C). Analysis calculated (%) for C_58_H_132_Cd_2_N_4_O_12_S_4_Si_4_: C 45.14, H 8.62, N 3.63, S 8.31; found: C 45.14, H 8.57, N 3.65, S 8.35. IR (solid state): ν 3331 (*w*), 3246 (*w*), 3161 (*vw*), 2971 (*vs*), 2930 (*m*), 2903 (*m*), 2858 (*w*), 1584 (*w*), 1471 (*w*), 1418 (*vw*), 1386 (*m*), 1361 (*s*), 1339 (*vw*), 1311 (*vw*), 1285 (*vw*), 1266 (*vw*), 1231 (*m*), 1210 (*m*), 1175 (*s*), 1137 (*w*), 1120 (*w*), 1091 (*vw*), 1083 (*vw*), 1036 (*vs*), 1012 (*s*), 1006 (*vs*), 998 (*vs*), 993 (*vs*), 974 (*m*), 952 (*m*), 912 (*w*), 882 (*vw*), 858 (*vw*), 813 (*m*), 793 (*w*), 734 (*vw*), 723 (*vw*), 688 (*m*), 677 (*m*), 657 (*m*), 607 (*vw*), 535 (*m*), 502 (*w*), 479 (*w*), 470 (*w*), 422 (*w*). ^1^H NMR (CH_3_OH-*d*_4_): δ 4.88 and 3.33 (methanol protons), 2.77 (*t*, 2H, *J*_HH_ = 6.9 Hz), 2.49 (*t*, 2H, *J*_HH_ = 7.2 Hz) and 1.73 (*q*, 2H, *J*_HH_ = 6.8 and 7.1 Hz) – methyl­ene protons of BAPP; 2.62 (*br m*, NH_2_ protons of BAPP); 1.42 (*s*, 54H, *t*Bu protons).

#### [Cd{SSi(O*t*Bu)_3_}_2_(μ-BAPP)]_*n*_, 2

[Cd{SSi(O*t*Bu)_3_}_2_]_2_ (0.1 mmol, 0.13 g) in toluene (8 ml) was mixed with BAPP (0.082 ml, 0.4 mmol) dissolved in methanol (2 ml). After gentle stirring, the mixture was allowed to stand for a few days at −20 °C. The obtained white precipitate was filtered off and recrystallized from toluene. Colourless crystals of **2** were obtained after two weeks of crystallization (yield 54%; m.p. 118.4–119.7 °C). Analysis calculated (%) for C_34_H_78_CdN_4_O_6_S_2_Si_2_: C 46.85, H 9.02, N 6.43, S 7.36; found: C 46.71, H 9.14, N 6.44, S 7.12. IR (solid state): ν 3293 (*m*), 3227 (*w*), 3152 (*w*), 2968 (*vs*), 2944 (*m*), 2934 (*m*), 2925 (*m*), 2872 (*w*), 2820 (*m*), 2778 (*w*), 2743 (*vw*), 2723 (*vw*), 2706 (*vw*), 2673 (*vw*), 1590 (*w*), 1492 (*vw*), 1458 (*w*), 1449 (*w*), 1383 (*m*), 1361 (*s*), 1347 (*w*), 1310 (*vw*), 1301 (*w*), 1263 (*w*), 1251 (*w*), 1239 (*m*), 1204 (*m*), 1185 (*s*), 1146 (*w*), 1128 (*w*), 1104 (*w*), 1074 (*w*), 1069 (*w*), 1040 (*vs*), 1004 (*vs*), 987 (*vs*), 961 (*m*), 940 (*w*), 910 (*w*), 882 (*vw*), 842 (*vw*), 820 (*m*), 802 (*m*), 770 (*w*), 728 (*vw*), 683 (*m*), 650 (*s*), 544 (*m*), 500 (*w*), 485 (*w*), 477 (*w*), 461 (*w*), 427 (*vw*), 422 (*w*). ^1^H NMR (toluene-*d*_6_): δ 2.54 (*br t*, 4H, methyl­ene protons of BAPP), 2.45 (*br m*, 4H, NH_2_ protons of BAPP), 2.17 (*t*, *J*_HH_ = 6.4 Hz, 4H, methyl­ene protons of BAPP), 1.38 (*s*, 54H, *t*Bu protons), 1.26 (*br m*, 4H, methyl­ene protons of BAPP); about 0.9 (very *br m*, 4H of methyl­ene protons of BAPP).

### X-ray crystallography

Crystal data, data collection and structure refinement details are summarized in Table 1 ▸. H atoms bonded to C atoms were refined using a riding model. The *U*_iso_(H) values of the methyl H atoms were set to 1.5*U*_eq_(C), while the *U*_iso_(H) values of the H atoms bonded to the remaining C atoms were set to 1.2*U*_eq_(C). All H atoms bonded to N atoms were refined freely. The structure of **1** contained a high residual electron-density peak located near atoms S1, S2 and N2. Both S atoms in **1** were refined as positionally disordered over two positions using the PART 1 and PART 2 commands (occupancies 0.79 and 0.21) and the data was calculated with 21.000 and −21.000.

### DFT calculations and NCI analysis

All quantum mechanical calculations were performed with the help of the supercom­puters of the Centre of Informatics Tricity Academic Supercom­puter & Network Academic Computer Center (TASK, Gdańsk, Poland) with *GAUSSIAN2016* software (Frisch *et al.*, 2016 ▸). The structures of the Cd com­plexes obtained from the diffraction studies were optimized at the M-11L/def2SV(P) level of theory (Peverati *et al.*, 2012 ▸; Weigend *et al.*, 2005 ▸) with an ultrafine grid and tight convergence criteria. IR frequencies were calculated with the same level of theory and none of these optimized structures exhibited negative frequencies. The selected M-11L functional is optimal for transition metals, organometallic com­pounds and the determination of noncovalent inter­actions (NCIs), while the selected basis set provides qualitatively accurate results within a reasonable calculation time. Larger basis sets were tested but turned out to be inefficient for such large mol­ecular com­plexes (over 200 atoms). Electron densities, a by-product of every density functional theory (DFT) calculation, have been saved to external files and utilized in the next step of the data analysis. The NCI analysis was performed with the *Multiwfn* software (Version 3.6; Lu *et al.*, 2012 ▸). The analysis was performed for the direct crystallographic structures of the Cd com­plexes and for their DFT-optimized coun­ter­parts. The electron densities of the former were calculated during a Single Point Energy calculation job type [M-11L/def2SV(P), without geometry optimization], while for the latter, they were calculated at the optimization step. Although, in the first case, electron densities were calculated without any optimization step, these results were used to com­pare how both experimental and DFT-optimized structures differed or not. The number of visualization points was 27 × 10^6^ or higher. Such a large number provided a clear and unscattered weak inter­action picture in each case. The *VMD* mol­ecular visualization program (Version 1.9.2) was used for visualization of the NCI results (Humphrey *et al.*, 1996 ▸). For better clarity, these inter­actions were excluded from the results, according to the *Multiwfn* manual, and only Cd–ligand or ligand–ligand inter­actions were visualized.

### Anti­microbial activity

The preliminary examination of the anti­fungal activity of com­pound **1** was performed with the microdilution plate method. RPMI 1640 supplemented with glucose and 3-(*N*-morpholino)­propane­sulfonic acid (MOPS) at a final concentration of 0.165 mol l^−1^ and pH 7.0 was applied as a culture medium. 18 isolates of fungi from the collection of the Department of Mol­ecular Biotechnology and Microbiology were examined, *i.e. Alternaria alternata*, *Aspergillus flavus*, *A. fumigatus*, *A. niger*, *Candida albicans*, *C. catenulata*, *C. haemulonii*, *C. glabrata*, *C. kefyr*, *C. krusei*, *C. parapsilosis*, *C. tropicalis*, *C. utilis*, *Fusarium oxysporum* and *F. solani*, and the three dermato­phyte isolates *Epidermophyton floccosum*, *Microsporum canis* and *Trichophyton rubrum*. All of the isolates were identified by conventional and mol­ecular identification prior to deposition in the collection. The inoculum was prepared by suspending one colony from Sabouraud agar in 3 ml of distilled water. The inoculum was ready after the density reached 0.5 according to the McFarland standard, which is equal to 1–5 × 10^6^ CFU ml^−1^. 100 µl of the suspension was transferred to each well of columns 1–9 of the microdilution plate with 100 µl twofold dilutions of com­pound **1** (ranging from 4 to 0.016 mg l^−1^). The wells in column 10 containing methanol were designed to exclude its influence, as **1** was dissolved in methanol. The wells in column 11 containing 100 µl of sterile drug-free medium containing pure inoculum served as the positive and sterility controls, and the wells in column 12 not containing inoculum served as the negative control. The microdilution plates were incubated for 24 h at 37 °C. The results were judged by eye after 24 h. The lowest concentration of **1** giving any inhibition of growth was regarded as the MIC value.

## Results and discussion

### Synthesis

We have examined the coordination abilities of 1,4-bis­(3-amino­prop­yl)pi­per­a­zine (BAPP) in reactions with Cd^II^ tri-*tert*-but­oxy­silane­thiol­ate, [Cd{SSi(O*t*Bu)_3_}_2_]_2_, under atmospheric conditions. At first, we combined [Cd{SSi(O*t*Bu)_3_}_2_]_2_ with BAPP in a molar ratio of 1:4 in methanol. The reaction yielded colourless crystals of dinuclear [Cd_2_{SSi(O*t*Bu)_3_}_4_(μ-BAPP)], **1**, isolated after several days of crystallization at 4 °C (Scheme 1). The reaction of [Cd{SSi(O*t*Bu)_3_}_2_]_2_ with BAPP at the same molar ratio but in a toluene–methanol solvent system also yielded a colourless precipitate of the polynuclear com­pound [Cd{SSi(O*t*Bu)_3_}_2_(μ-BAPP)]_*n*_, **2**, which was further recrystallized from toluene at low temperature (−20 °C). After two weeks, colourless crystals of **2** suitable for X-ray analysis were obtained (Scheme 1). To check whether the solvent for the recrystallization influences the final structure of the product, we recorded ^1^H NMR spectra for the precipitate obtained before recrystallization. The results clearly indicate that there are no free ligands in the precipitated product of the reaction before recrystallization (Figs. S1–S4 in the supporting information). Thus, one can assume that the use of toluene is crucial for obtaining polynuclear com­plex **2**. The obtained com­pounds were synthesized in fairly high yields and were stable under atmospheric conditions, enabling further tests to be carried out to determine their physicochemical properties.

### Crystal structures

**Figure 1:**
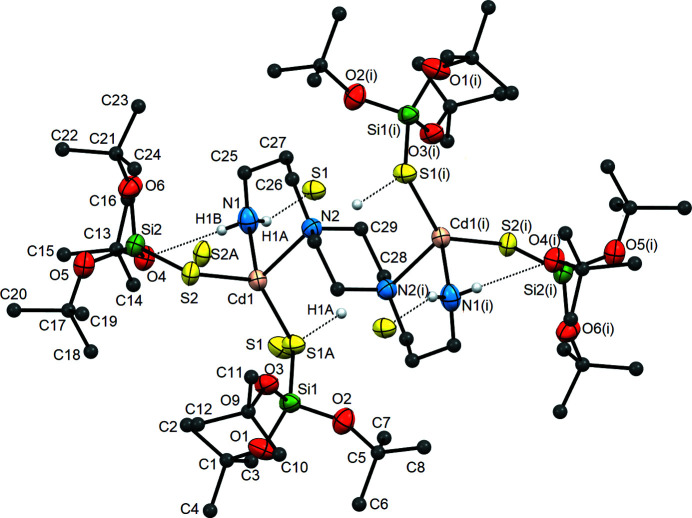
A fragment of the crystal structure of **1**, showing the environments of the metal centres, the atom-labelling scheme for the asymmetric unit and weak hydrogen bonding (as dashed lines). Displacement ellipsoids are drawn at the 50% probability level and the H atoms of the *tert*-butyl groups and of the BAAP ligand bonded to C atoms have been omitted for clarity. [Symmetry code: (i) −*x* + 2, −*y* + 1, −*z* + 1.]

X-ray analysis results have shown that com­plexes **1** and **2** crystallize in the monoclinic space group *P*2_1_/*n*. Complex **1** is dinuclear with metallic centres connected *via* the mol­ecule of BAPP, which acts as a tetra­dentate bridging ligand and leads to the formation of a dimeric com­pound (Fig. 1 ▸ and Fig. S5 in supporting information). Each of the Cd^II^ atoms in com­pound **1** is chelated by two N atoms, *i.e.* one from the BAPP ring and the second from the amine group of the amino­propyl residues. The tetra­hedral coordination sphere of each Cd^II^ atom is com­pleted by two tri-*tert*-but­oxy­silane­thiol­ate residues acting as *S*-donor terminal ligands.

The bond angles around the Cd^II^ atoms are in the range 88.42 (11)–135.39 (13)° (Table S1), indicating the presence of slight deviations from tetra­hedral geometry, which was confirmed by the values of the structural parameters τ_4_ and τ_4_′ (0.77 and 0.70, respectively) (Fig. S7) (Yang *et al.*, 2007 ▸; Okuniewski *et al.*, 2015 ▸; Rosiak *et al.*, 2018 ▸). However, these deviations do not affect the Cd—S bond lengths, while one of the Cd—N bonds is slightly longer when com­pared to those observed in other cadmium(II) silane­thiol­ates with a tetra­hedral metallic centre (Table S2) (Dołęga *et al.*, 2006 ▸, 2007 ▸; Pladzyk *et al.*, 2013 ▸, 2015 ▸; Kowalkowska *et al.*, 2017 ▸; Maślewski *et al.*, 2017 ▸). The mol­ecule of **1** is centrosymmetric, with the inversion centre located in the middle of the pi­per­a­zine ring of the BAPP mol­ecule. The duplication of the asymmetric unit of com­plex **1** by this centre generates the second part of the dimer.

BAPP mol­ecules are involved in the formation of hydrogen bonds, *i.e.* intra­molecular N1(BAPP)—H1*B*⋯O4(TBST) [*D*⋯*A* = 3.115 (4)Å] and inter­molecular N1(BAPP)—H1*A*⋯S1(TBST) [*D*⋯*A* = 3.519 (5) Å]. The parameters of these inter­actions are presented in Table S3. The presence of these inter­molecular inter­actions causes the individual mol­ecules of com­pound **1** to be inter­connected and form one-dimensional chain structures through *R* 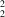 rings that join neighbouring molecules (Bernstein *et al.*, 1995 ▸). The distances between the Cd^II^ atoms in the mol­ecule of **1** and the distances to the metallic centres of neighbouring mol­ecules are almost identical at 6.3474 (6) and 6.3324 (6) Å, respectively.

**Figure 2:**
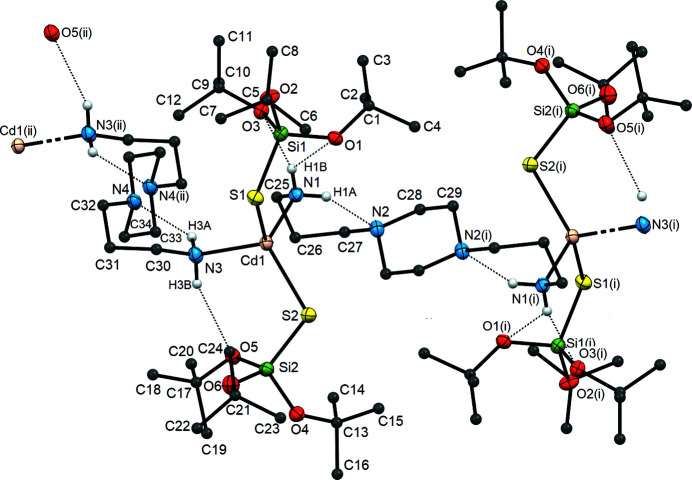
A fragment of the crystal structure of **2**, showing the environments of the metal centres, the atom-labelling scheme for the asymmetric unit and weak hydrogen bonding (as dashed lines). Displacement ellipsoids are drawn at the 50% probability level and the H atoms of the *tert*-butyl groups and of the BAAP ligand bonded to C atoms have been omitted for clarity. [Symmetry codes: (i) −*x* + 1, −*y*, −*z* + 1; (ii) −*x* + 1, −*y* + 1, −*z* + 1.]

Compound **2** is a polynuclear com­plex with a polymeric structure (Fig. 2 ▸ and S6). As in com­plex **1**, each Cd^II^ atom is coordinated by two S atoms from TBST residues and two N atoms from the *R*NH_2_ groups of two BAPP bridging ligands that connect adjacent metallic centres.

The Cd—N and Cd—S bond lengths observed in **2** are similar to those found in other heteroleptic cadmium(II) silane­thiol­ates (Table S1) (Dołęga *et al.*, 2006 ▸, 2007 ▸; Pladzyk *et al.*, 2013 ▸, 2015 ▸; Kowalkowska *et al.*, 2017 ▸; Maślewski *et al.*, 2017 ▸), whereas the angles at the Cd^II^ atoms range from 99.26 (10) to 120.28 (3)°. This indicates the presence of even smaller deviations from ideal tetra­hedral geometry com­pared to com­plex **1** (τ_4_ = 0.91 and τ_4_′ = 0.88) (Fig. S8 and Table S2) (Yang *et al.*, 2007 ▸; Okuniewski *et al.*, 2015 ▸; Rosiak *et al.*, 2018 ▸). Coordination polymer **2** is also centrosymmetric, with an inversion centre located at the middle of the six-membered pi­per­a­zine ring of BAPP. The distances between the nearest Cd^II^ atoms within a single chain are 10.222 (2) and 11.424 (2) Å, while the distances between atoms belonging to neighbouring chains are shorter at 8.949 (2) and 9.677 (3) Å. The spatial arrangement of the polymeric chains of **2** enables the formation of diverse intra­molecular inter­actions. Atoms N1 and N3 of the amino groups of the BAPP mol­ecules are donors of two types of hydrogen-bonding inter­actions (Fig. 2 ▸). The first type is N(BAPP)—H⋯N(BAPP ring), between the amino group and an N atom belonging to the pi­per­a­zine ring from the same BAPP ligand, *i.e.* N1—H1*A*⋯N2 [*D*⋯*A* = 2.977 (4) Å], as well as N3—H3*A*⋯N4 [*D*⋯*A* = 2.996 (4) Å]. The second type is the N(BAPP)—H⋯O(TBST) hydrogen-bonding inter­action between the same amino group and the O atom of the silane­thiol­ate residue, *i.e.* N1—H1*B*⋯O3 [*D*⋯*A* = 3.200 (4) Å] (Table S3). In addition, atom N1 is a donor in the next inter­action, N1—H1*B*⋯O1, with the O atom of the TBST residue as the acceptor, so that the H1*B* atom is engaged in the formation of a three-centred hydrogen bond. The crystal packing of **2** shows that the polymer chains are arranged parallel to each other with no further significant inter­actions.

### FT–IR spectroscopy

The FT–IR spectra were recorded for both com­plexes in the solid state (Fig. S9 in the supporting information). They are consistent with the crystal structures and confirm the presence of the ligands used in the syntheses (Nakamoto, 1997 ▸). The spectra of **1** and **2** contain bands of various intensities in the range from 3161 to 2675 cm^−1^, characteristic for the symmetric and asymmetric vibrations of the C—H bonds of the methyl­ene groups of BAPP, as well as the methyl groups present in the TBST residues. The number of bands occurring in this range for com­pound **2** is greater than for com­plex **1**. The bands observed in the range 1100–980 cm^−1^ for both com­plexes are characteristic for the Si—O—C bonding present in the (*t*BuO)_3_SiS^−^ residues, and their patterns are typical for silane­thiol­ate residues coordinating to metallic centres as terminal *S*-donor residues (Pladzyk *et al.*, 2021 ▸).

The BAPP ligand may be identified by the presence of the N—H symmetric and asymmetric stretching vibrations present at about 3330 cm^−1^ (3332 and 3257 cm^−1^ for **1**, and 3293 and 3232 cm^−1^ for **2**), the N—H deformation at 1584 and 1590 cm^−1^ for **1** and **2**, respectively, as well as the in-plane and out-of-plane N—H vibrations in the ranges 1480–1440 (1481 cm^−1^ for **1**, and 1492, 1458, and 1449 cm^−1^ for **2**) and 800–780 cm^−1^, respectively. Other peaks typical for cyclic amines are present at 1266, 1238 and 1204 cm^−1^ for **1**, and at 1263, 1251 and 1240 cm^−1^ for **2**, and correspond to C—N vibrations (Prabavathi *et al.*, 2015 ▸).

The def2sv(p) functional was applied in *GAUSSIAN16* (Frisch *et al.*, 2016 ▸) for the calculation of the FT–IR spectra of **1** and **2**. A com­parison of the experimental and calculated spectra was carried out, together with assignments of the important features concluded from calculations (Table S4 and Fig. S10). Both the experimental and theoretical IR spectra are remarkably consistent. Virtually all vibrations in the experimental spectra can be assigned with the help of the calculated spectra, which confirms that the synthesis of both com­pounds was successful. Although no IR scale factor for the M11-L/def2sv(p) level of theory was found in the literature, it can be estimated as 0.92–0.93 on the basis of the N—H stretching bands, and mainly concerns higher wavenumber bands (about 3000 cm^−1^).

The main difference between the experimental spectra of **1** and **2** is the additional N—H stretching band in **2**. This phenomenon is also reflected in the theoretical spectra. The reason for this is the inequality of various parts of the BAPP mol­ecule in **2** (see Section 3.4). The inequality of the environments of the BAPP end groups in **2** allows for distinguishable differences in the N—H bond energies and vibration frequencies. Meanwhile, in **1**, both ends of the mol­ecule inter­act symmetrically with two cadmium centres, *i.e.* they are equal or almost similar in energy and frequency.

### NCI analysis of 1 and 2

The optimization step of the DFT calculations slightly altered the geometry of the initial com­plexes, and the overall structures of the crystallographic and optimized com­plexes were very similar. NCI inter­action sites were initially determined for both the crystallographic and the DFT-optimized structures of com­plexes **1** and **2** within 1 nm of the Cd atom to verify if the crystallographic structure could be used directly in the qualitative NCI analysis. The optimized structures exhibited virtually all the inter­action sites and properties present in the crystallographic structures; thus, the NCI analysis was performed for the real crystallographic structures. The IR spectra were derived from the optimized structures of both com­plexes.

Usually, NCI analysis gives information on the type and strength of a weak bond. In this group of inter­actions, hydrogen bonds are considered the strongest visible in the NCI analysis results and are usually depicted as well-defined blue disks, according to the convention of the *Multiwfn* software and the developers of the method (Lu *et al.*, 2012 ▸). The disk shape reveals its directionality and the darker the blue colour, the stronger the bond. It should be noted that most Cd coordination bonds are stronger than an average hydrogen bond and NCI analysis indicates them not as blue disks (as for strong hydrogen bonds), but as blue/red rings. The hollow centre of such visualizations indicates that their classification as generally weak noncovalent inter­actions is barely justified.

#### Weak inter­actions in the vicinity of Cd

**Figure 3:**
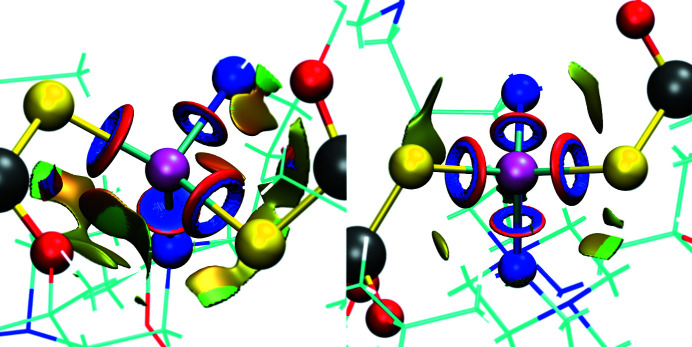
Weak inter­actions in the vicinity of the Cd atom as a result of the NCI analysis, with **1** on the left and **2** on the right. Green/olive irregular patches denote weak van der Waals-type inter­actions, while blue/red disks or rings denote relatively strong direct coordination inter­actions.

The neighbourhoods of the Cd atoms in **1** and **2** are strikingly different (see Fig. 3 ▸). These differences may be directly recognizable in these structures, yet NCI results aid in fully understanding them. The four main inter­action sites in **2** are highly symmetrical. Both N—Cd sites are strong and energetically very similar, which is reflected in their almost identical shape and colour of the inter­action indicator (blue/red ring). The same can be said for the S—Cd inter­actions. The overall environment of the Cd centre is open and accessible for other possible inter­actions.

Meanwhile, the N—Cd or S—Cd inter­actions in **1** are not equal. In particular, one of the N—Cd inter­actions turns out to be significantly weaker than the analogous inter­action in **2** (full blue/red disk between Cd and N). The symmetry of the inter­actions is broken in com­parison to **2**, probably due to the fact that both N—Cd inter­actions come from a single BAPP mol­ecule, *i.e.* three mol­ecules contribute to four Cd inter­actions. In the case of **2**, all four Cd coordination inter­actions are formed by four different mol­ecules, which allows for more freedom in the spatial orientation of the ligand sites and is closer to the tetra­hedral coordination of Cd.

The overall contribution of the van der Waals inter­actions (green/olive patches) in the case of **1** is significantly higher. The Cd atom in this com­plex is densely covered with other atoms of the structure, leaving no place for other inter- or intra­molecular inter­actions. In particular, the spatial orientation of S—Si—O allows for weak O—Cd inter­actions in **1** (green patches between Cd and O), which is not possible in **2**. Most probably, these inter­actions are possible due to the inequality of the N—Cd inter­actions and the greater deviation of the Cd inter­action distribution from the optimal tetra­hedral orientation.

#### Weak inter­actions of 1,4-bis­(3-amino­prop­yl)pi­per­a­zine (BAPP)

The better packing of **1** is also reflected in the number of inter­actions with the BAPP ligand. A visual inspection of the NCI results (see Figs. S11–S14 in the supporting information), focused this time on inter­molecular inter­actions of the ligand with the rest of the structures of **1** or **2**, gives the impression that tightly organized com­plex **1** takes advantage of almost all possible inter­molecular inter­actions involving Cd and the BAPP and TBST ligands. Van der Waals inter­actions are numerous and relatively large in **1**, while in **2**, a scattered pattern of these weak inter­actions is revealed (in both possible variants of the ligand). Even though all inter­actions other than N—Cd are weak, their co-operativity should make the structure of **1** much more stable and stronger than that of **2**.

Moreover, weak but well-oriented C—H hydrogen bonds, symbolized by small green disks in the axis of C—H bonds, may play a significant role in the maintenance of the densely packed crystal structure. Eight such inter­actions can be spotted in the case of the BAPP fragment in **1**, while only two such directional inter­actions can be recognized in both variants of the ligand in **2**.

It can be argued that these kinds of inter­actions in **1** can be called true hydrogen bonds, yet such a directional and in-axis character, supported by a relatively large number per BAPP mol­ecule, makes them stronger anyway.

### Luminescence

The solid-state luminescence properties of powder samples of **1** and **2**, together with the free BAPP ligand and [Cd{SSi(O*t*Bu)_3_}_2_]_2_, were investigated at ambient temperature. The entire spectroscopic study was carried out under identical experimental conditions. The optical absorption spectra of **1** and **2** have been measured by diffuse-reflectance experiments (see Fig. S15). The diffuse-reflectance spectra show two sharp absorption bands in the UV region at 226 and 257 nm for **1**, and at 225 and 252 nm for **2**, with a weaker signal region at 280–380 nm. The observed bands in this region of the prepared com­pounds can be assigned to electronic transitions from the ground-state S_0_ level to the excited-state S_*n*_ levels of the BAPP pi­per­a­zine ligand. The diagram of the energy levels for com­plexes [pipH]_2_[Co(NCS)_4_] and [pipH]_2_[Ni(NCS)_4_] (where pip is pi­per­a­zine) has been proposed by Bie *et al.* (2005 ▸). Emission spectra were then recorded using these specific ultraviolet wavelengths (Fig. S16). In the case of **1**, a weak emission was observed, with a maximum located at about 350 nm, whereas in the case of **2**, week emissions located at about 350 and 420 nm were observed. This shows that excitation of the systems to the highest excited levels results in low emission intensities caused by large energy losses due to efficient non-radiative transitions to lower excited levels, from where emission takes place (Bie *et al.*, 2005 ▸).

**Figure 4:**
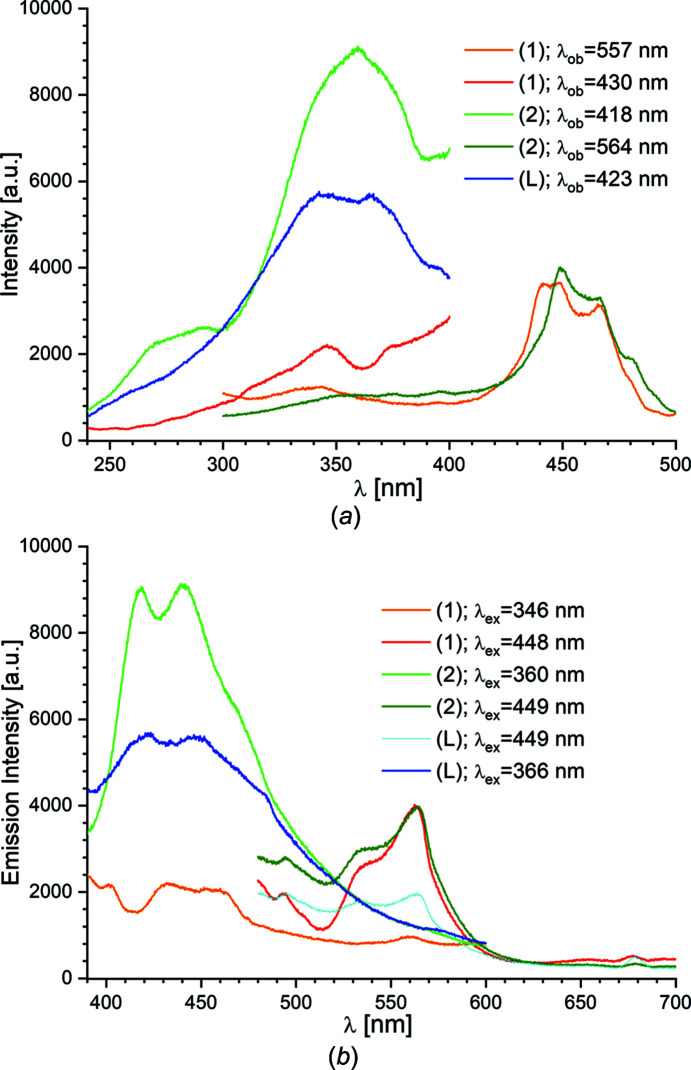
Room-temperature solid-state (*a*) excitation and (*b*) emission spectra of the BAPP ligand and com­pounds **1** and **2**.

Irradiation of all the systems with ultraviolet light in the solid state, monitoring the resulting emission at around 420 nm, was performed in order to determine the wavelength causing the greatest emission intensity. The excitation and emission spectra for the free BAPP ligand and com­pounds **1** and **2** are depicted in Fig. 4 ▸. Fig. 4 ▸(*a*) presents two excitation peaks of almost equal intensities (340 and 366 nm) for the free BAPP ligand. Upon incorporation of the BAPP ligand with Cd^II^ ions, excitations are observed at 346 and 370 nm for com­pound **1**, and at 360 nm for com­pound **2**. The maximum luminescence of the BAPP ligand is observed at 423 and 446 nm upon excitation at λ_ex_ = 366 nm, which corresponds to a blue luminescence. The shapes of the emission spectra of **1** and **2** are similar to the free BAPP emission. As seen in the figure, upon excitation at 346 nm, com­plex **1** exhibited weak emission peaks at 431 and 453 nm, which showed a small red shift of about 8 nm com­pared with the BAPP ligand. As in the case of the BAPP ligand, com­plex **2** shows a strong blue luminescence, with the main peaks at 418 and 439 nm, at an excitation wavelength of 360 nm. Unlike com­plex **1**, these emission bands are blue-shifted relative to the bands observed for the BAPP ligand. Broadband blue emission has been successfully realized in [pipH]_2_[Co(NCS)_4_] and [pipH]_2_[Ni(NCS)_4_] (Bie *et al.*, 2005 ▸), [Hg(μ_2_-*L*H)Cl_2_]_2_[Hg_2_(μ_2_-Cl)_2_Cl_4_]·2H_2_O and [Hg_4_(μ_3_-*L*)_2_(μ_2_-Cl)_2_Cl_6_] [*L* is *N*-(2-amino­eth­yl)pi­per­a­zine] (Li *et al.*, 2007 ▸), [(N-AEPz)ZnCl_4_]Cl (N-AEPz is *N*-amino­ethyl­pi­per­a­zine) (Zhang *et al.*, 2020 ▸), (CuI)_2_(*N*,*N*′-di­ethyl­pi­per­a­zine) (Safko *et al.*, 2012 ▸), (CuCN)_2_(Pip) and (CuCN)_2_(Me_2_Pip) (Me_2_Pip is *N*,*N*′-di­methyl­pi­per­a­zine) (Lim *et al.*, 2008 ▸), Ag(*L*)(ReO_4_) [*L* is *N-*(2-amino­eth­yl)pi­per­a­zine] (Kovalev *et al.*, 2015 ▸) and {[Cd(C_4_H_11_N_2_)(μ-Cl)_2_Cl]·H_2_O}_*n*_ (Mabrouk *et al.*, 2015 ▸). According to an earlier report, the free pi­per­a­zine ligand has an emission band at 418 nm on excitation at 312 nm (Bie *et al.*, 2005 ▸).

The BAPP luminescence emission position is red-shifted to 534 nm upon excitation at 449 nm and is accom­panied by the appearance of a green luminescence (Fig. 4 ▸). In com­parison with the blue luminescence, the green luminescence is characterized by its low intensity. Under 448 nm irradiation, com­plex **1** displayed a green emission with a peak at 562 nm. When **2** was excited at 449 nm, the luminescence spectrum exhibited an emission peak centred at 565 nm. However, com­pared to the BAPP ligand, the corresponding emission bands of com­pounds **1** and **2** have higher emission intensities. Considering the energy diagram proposed by Bie *et al.* (2005 ▸), the observed green emission is probably the result of a radiative transition from the lowest excited state of the ligand to its ground-state level.

Admittedly, the TBST residue present in **1** and **2** contains an Si—S bond where both elements have vacant 3*d* orbitals and, as a result, may participate in the emission process. Previous theoretical studies of the protonation and deprotonation of tri-*tert*-but­oxy­silane­thiol using DFT and natural bond orbital (NBO) calculations have shown that lone pairs from sulfur in Si—S bonds are delocalized due to inter­action with the anti­bonding σ*Si–O orbitals (Chojnacki, 2008*a* ▸,*b* ▸). Also, because of the polarization effects, these orbitals are strongly positioned on the silicon side. Therefore, the Si—S bonds are unlikely to condition the occurrence of emissions of com­plexes **1** and **2**. To confirm this, we conducted an additional experiment to determine whether the starting substrate [Cd{SSi(O*t*Bu)_3_}_2_]_2_ exhibits emission when excited and we ob­served no emission. Thus, we can assume that the luminescence differences between the emission properties of **1** and **2** (red and blue shifts relative to BAPP, and different intensities of their emission bands) should be attributed to their different structures, as shown by the X-ray studies.

### Thermal analysis

**Figure 5:**
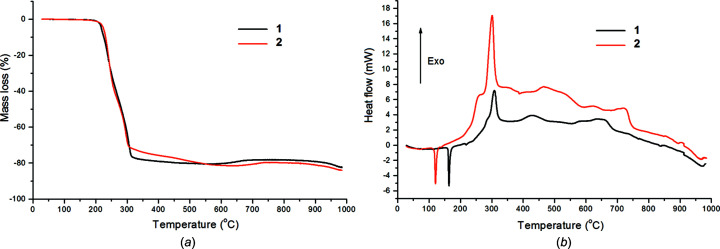
Thermal analysis of com­plexes **1** and **2** in air, showing (*a*) the TG curves and (*b*) the DSC curves.

The thermal behaviour of **1** and **2** was investigated simultaneously by TG–DSC (thermogravimetry–differential scanning calorimetry) and TG–FT–IR (thermogravimetry–Fourier transform infrared) methods. The cadmium com­plexes show different thermal stabilities under an air atmosphere. The dimeric com­plex **1** exhibits slightly lower thermal stability (177 °C) in com­parison with polymeric com­plex **2** (187 °C). This observation can be explained in terms of their different crystal structures. In dimeric com­plex **1**, the Cd^II^ atoms are chelated by the amine ligand, forming a six-membered ring, which slightly reduces the thermal stability of the metal com­plex. The decom­position processes of both com­plexes [Fig. 5 ▸(*a*)] are preceded by the melting processes observed on the DSC curves [Fig. 5 ▸(*a*)].

**Figure 6:**
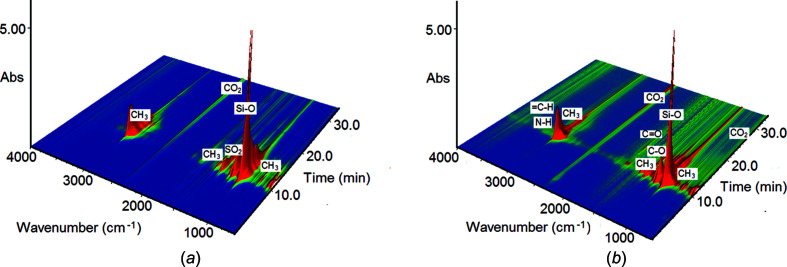
The FT–IR spectra of the evolved com­pounds during thermal decom­position of (*a*) **1** and (*b*) **2** under nitro­gen.

The endothermic effects associated with melting were observed at 163.1 and 120.2 °C (peak tops) for **1** and **2**, respectively. Further heating results in the decom­position of the com­plexes connected with the significant mass losses observed on the TG curves. The mass losses of 76.50 (for **1**) and 70.41% (for **2**) were found in the relatively narrow temperature ranges of 188–317 and 151–308 °C. A detailed analysis of the DSC curve of **1** allows, in the above temperature range, a very weak endothermic effect to be distinguished at 217 °C (peak top). The DSC curves of both com­plexes are dominated by strong exothermic effects, with a maximum at about 300 °C [Fig. 6 ▸(*a*)]. These effects can be attributed to the burning processes of the organic parts of the cadmium com­plexes. Further heating of the solid residues causes some mass changes connected with their transformations [Fig. 6 ▸(*a*)]. The above-mentioned distinct mass losses point most probably to the formation of cadmium thio­silicate (Cd_2_SiSO_3_). At higher temperatures, further mass losses of 3.50 (for **1**) and 10.45% (for **2**) are observed. Taking into account that these mass changes were accom­panied by exothermic effects, it can be assumed that the S atom of thio­silicate was oxidized and that the transformation of Cd_2_SiSO_3_ into Cd_2_SiO_4_ took place (Su *et al.*, 2018 ▸). Cadmium silicate is thermally stable in the tem­per­ature range 730–850 °C and, next, its further transformation takes place with the probable formation of cadmium oxide and silica (Kropidłowska *et al.*, 2007 ▸). The total observed mass losses recorded at 1000 °C for **1** and **2** were 82.42 and 84.00%, respectively.

Under a nitro­gen atmosphere, the thermal decom­position of both com­pounds occurs above 160 °C. The TG curves exhibit significant mass losses of 81.3 and 82.0% up to 300 °C for **1** and **2**, respectively. The total recorded mass loss at 700 °C was 85% for both metal com­plexes. The solid residues of the cadmium com­plexes heated under an inert atmosphere are com­posed of some unidentified cadmium com­pounds and unburnt carbon species.

The FT–IR spectra of the gaseous products of the thermal decom­position of the metal com­plexes are dominated by bands derived from the evolved Si–alk­oxy and *tert*-butyl com­pounds (Fig. 6 ▸). A very strong band at 1070 cm^−1^ was assigned to the stretching vibrations of the Si—O groups. The several bands with the strongest maxima at 2980 and 2942 cm^−1^, as well as those at 1457 cm^−1^, can be ascribed to the stretching and deformation vibrations of the methyl groups from the evolved moieties. The band at 1188 cm^−1^ can be assigned to the stretching vibrations of the C—O group from the tertiary alcohol mol­ecules (Holly *et al.*, 1975 ▸; Silverstein & Webster, 1996 ▸). Breaking of the Si—S bonds in the mol­ecules of the investigated cadmium com­plexes leads to the evolution of sulfur dioxide, which gives diagnostic double bands at 1390 and 1367 cm^−1^ (Łyszczek *et al.*, 2015 ▸). Heating of the com­plexes under an inert atmosphere also leads to the evolution of aliphatic hydro­carbons as a result of degradation of the coordinated amine group. The presence of bands in the regions 3000–2700 and 1480–1360 cm^−1^, as well as those at 860 and 821 cm^−1^, due to stretching, deformation and rocking vibrations of methyl groups, can indicate the evolution of the ethane/propane mol­ecules. The IR spectra of com­plex **2** shows a broad weak band in the region 3200–3000 cm^−1^, with a maximum at 3080 cm^−1^, and a medium intensity band at 1277 cm^−1^, which can be ascribed to the C—H, =C—H and C—N stretching vibrations of some aliphatic amines and/or alkenes (Holly *et al.*, 1975 ▸; Silverstein & Webster, 1996 ▸). The formation of volatile species containing carbonyl groups (C=O) during the thermal decom­position of **2** is postulated based on the presence of a band at 1773 cm^−1^. The FT–IR spectra of volatile com­pounds recorded above 400 °C also show relatively weak bands at 2341/2341 and 689 cm^−1^ from stretching and deformation vibrations of carbon dioxide mol­ecules. It is worth mentioning that the intensity of CO_2_ evolution is greater for com­pound **2** (Fig. 6 ▸).

### Anti­microbial activity

The effective concentration of com­plex **1** was checked from 4 to 0.016 mg l^−1^. The performed susceptibility tests exhibited a lack of anti­fungal activity of **1** against 14 of the tested isolates, but for three tested dermato­phytes, *i.e. Epidermophyton floccosum*, *Microsporum canis* and *Trichophyton rubrum*, the inhibition of their growth was observed at 0.25 mg l^−1^ and above. The mechanism of the toxicity of **1** towards the fungal isolates relies on Cd, however, the influence of other groups cannot be excluded thus far. Moreover, the fungal specimens are generally considered to be tolerant to heavy metals due to their presence in soil (Rajapaksha *et al.*, 2004 ▸; Bhajbhuje *et al.*, 2013 ▸; Li *et al.*, 2019 ▸). In the case of the influence of cadmium on the dermato­phytes, there has been one report confirming its anti­fungal activity; however, the test was performed with the colony diameter method (Al-Janabi, 2011 ▸). This specific anti­fungal activity may also be related to the fact that the development and growth of dermato­phytes depends strongly on keratin, a hydro­phobic protein rich in sulfur-containing amino acids such as cysteine and me­thio­nine (Ciesielska *et al.*, 2021 ▸), which, being soft bases, have a particular affinity for soft acids like Cd^2+^ ions. To address the question of the anti­dermato­phyte effect of **1**, further investigations are being considered.

## Conclusion

In summary, this study has provided detailed insight into the structures of two new heteroleptic cadmium tri-*tert*-but­oxy­silane­thiol­ates with 1,4-bis­(3-amino­prop­yl)pi­per­a­zine synthesized in different solvent systems while maintaining the remaining reaction conditions. The experiments yielded com­pounds with different structures and the absence of solvent mol­ecules crystallizing in the structures. The structures of the obtained com­plexes appeared to be crucial for their spectral properties and anti­fungal activity. Com­pound **1** inhibits the growth of fungi belonging to the group of dermato­phytes. The results of our study show that it is worth extending the study to other metal silane­thiol­ates with the purpose of obtaining com­pounds with better anti­microbial activity and luminescence.

## Supplementary Material

Crystal structure: contains datablock(s) dkz2, dkz1, global. DOI: 10.1107/S2053229623005442/dv3023sup1.cif

Structure factors: contains datablock(s) dkz2. DOI: 10.1107/S2053229623005442/dv3023dkz2sup3.hkl

Structure factors: contains datablock(s) dkz1. DOI: 10.1107/S2053229623005442/dv3023dkz1sup2.hkl

Additional tables and figures. DOI: 10.1107/S2053229623005442/dv3023sup4.pdf

CCDC references: 2192696, 2192695

## Figures and Tables

**Table 1:** Experimental details For both structures: monoclinic, *P*2_1_/*n*. Experiments were carried out at 120 K with Mo *K*α radiation using a Stoe IPDS 2T diffractometer. H atoms were treated by a mixture of independent and constrained refinement.

	Monomer **1**	Polymer **2**
Crystal data		
Chemical formula	[Cd_2_(C_12_H_27_O_3_SSi)_4_(C_10_H_24_N_4_)]	[Cd(C_12_H_27_O_3_SSi)_2_(C_10_H_24_N_4_)]
*M*_r_	1543.07	871.7
*a*, *b*, *c* (Å)	9.6433 (2), 29.0546 (4), 14.5665 (2)	16.424 (5), 16.876 (4), 18.265 (5)
β (°)	91.466 (1)	112.66 (2)
*V* (Å^3^)	4079.94 (12)	4672 (2)
*Z*	2	4
μ (mm^−1^)	0.73	0.65
Crystal size (mm)	0.44 × 0.26 × 0.14	0.38 × 0.34 × 0.3

Data collection		
Absorption correction	Multi-scan [*LANA* (Koziskova *et al.*, 2016 ▸) in *X-AREA* (Stoe & Cie, 2016 ▸)]	Multi-scan [*LANA* (Koziskova *et al.*, 2016 ▸) and *X-RED32* in *X-AREA* (Stoe & Cie, 2016 ▸)]
*T*_min_, *T*_max_	0.409, 1.000	0.664, 0.970
No. of measured, independent and observed [*I* > 2σ(*I*)] reflections	39464, 8337, 7133	33547, 9535, 8345
*R*_int_	0.028	0.045
(sin θ/λ)_max_ (Å^−1^)	0.625	0.625

Refinement		
*R*[*F* ^2^ > 2σ(*F* ^2^)], *wR*(*F* ^2^), *S*	0.044, 0.123, 1.04	0.043, 0.107, 1.14
No. of reflections	8337	9535
No. of parameters	424	476
Δρ_max_, Δρ_min_ (e Å^−3^)	1.52, −0.71	1.32, −0.74

Computer programs: *WinXpose*, *Recipe*, *Integrate* and *X-RED32* in *X-AREA* (Stoe & Cie, 2016 ▸), *SHELXT2014* (Sheldrick, 2015*a* ▸), *SHELXL2018* (Sheldrick, 2015*b* ▸), *ORTEP-3 for Windows* (Farrugia, 2012 ▸), *Mercury* (Macrae *et al.*, 2020 ▸), *WinGX* (Farrugia, 2012 ▸) and *OLEX2* (Dolomanov *et al.*, 2009 ▸).
