# Supplementary material for: Solvent influence on the crystal structures of new cadmium tri-tert-but­oxy­silane­thiol­ate com­plexes with 1,4-bis­(3-amino­prop­yl)pi­per­a­zine: luminescence and anti­fungal activity
Source: Acta Crystallogr C Struct Chem. 2023 Jul 5;79(Pt 8):305–15. doi: 10.1107/S2053229623005442 (PMC10404125; doi:10.1107/S2053229623005442)
Supplement: Supplementary file 4 [file c-79-00305-sup4.pdf]

STRUCTURAL CHEMISTRY

Volume 79 (2023)

Supporting information for article:

Solvent influence on the crystal structures of new Cd tri-*tert*-butoxysilanethiolate complexes with 1,4-bis(3-aminopropyl)piperazine: luminescence and antifungal activity

Daria Kowalkowska-Zedler, Piotr Bruździak, Zbigniew Hnatejko, Renata Łyszczek, Anna Brillowska-Dąbrowska, Łukasz Ponikiewski, Bartosz Cieśla and Agnieszka Pladzyk

S1. Structural data

Table S1 Selected bond angles (°) for complexes **1** and **2**.

Bond angles [°]	**1**	**2**
N1—Cd1—N2	88.42(11)	
N1—Cd1—N3		99.26(10)
N1—Cd1—S1	103.01(15)	106.89(8)
N1—Cd1—S1A	98.2(5)	
N1—Cd1—S2	107.32(14)	111.36(8)
N1—Cd1—S2A	99.7(8)	
N2—Cd1—S1	116.12(19)	
N2—Cd1—S1A	124.5(7)	
N2—Cd1—S2	96.83(13)	
N2—Cd1—S2A	86.5(12)	
N3—Cd1—S1		107.26(8)
N3—Cd1—S2		109.68(8)
S1—Cd1—S2	135.39(13)	120.28(3)
S1—Cd1—S2A	148.0(13)	
S1A—Cd1—S2A	144.5(11)	
Si1—S1—Cd1	103.74(19)	104.26(5)
Si1—S1A—Cd1	95.6(9)	
Si2—S2—Cd1	103.75(19)	110.61(5)
Si2—S2A—Cd1	107.0(5)	

Table S2 Selected bond distances (Å) for complexes **1** and **2.**

Bond length [Å]	**1**	**2**
Cd1—N1	2.310(3)	2.301(3)
Cd1—N2	2.415(3)	
Cd1—N3		2.301(3)
Cd1—S1	2.428(4)	2.4675(12)
Cd1—S1A	2.56(2)	
Cd1—S2	2.468(5)	2.4597(10)
Cd1—S2A	2.309(10)	
Si1—S1	2.068(4)	2.0805(13)
Si1—S1A	2.22(2)	
Si2—S2	2.077(3)	2.0773(13)
Si2—S2A	2.146(18)	
Si1—O1	1.603(3)	1.630(2)

Si1—O2	1.635(3)	1.627(2)
Si1—O3	1.632(2)	1.639(2)
Si2—O4	1.636(2)	1.625(2)
Si2—O5	1.629(3)	1.647(2)
Si2—O6	1.626(3)	1.628(2)

Table S3 Hydrogen bonds parameters for complexes **1** and **2**.

	D—H···*A*	*D*—H [Å	H···*A* [Å]	*D*···*A* [Å]	∠*DHA*
**1**	N1—H1B···O4	0.84(5)	2.41(5)	3.115(4)	142(4)
	N1—H1A···S1[a]	0.86(5)	2.70(5)	3.519(5)	159(4)
**2**	N1—H1A···N2	0.90(4)	2.24(4)	2.977(4)	139(3)
	N3—H3A···N4	0.96(4)	2.23(4)	2.996(4)	136(3)
	N1—H1B···O3	0.85(4)	2.39(4)	3.200(4)	160(3)
	N3—H3B···O5	0.90(5)	2.42(5)	3.256(4)	154(4)

Symmetry code(s): (*a*) –x+1, –y+1, –z+1;

Table S4 Experimental and def2sv(p) calculated frequencies, and general assignments for observed IR bands in the FT-IR spectrum of **1** and **2** (stretching frequencies and fingerprint region). No scaling to the calculated spectrum was applied.

Compound **1**			Compound **2**		
Experimental [cm^{-1}]	Calculated [cm^{-1}]	Assignment from calculations	Experimental [cm^{-1}]	Calculated [cm^{-1}]	Assignment from calculations
3331 (w)	3610	$–NH_2$ asym. stretch	3293 (m)	3550<br>3536	$–NH_2$ asym. stretch
3246 (w)	3501	$–NH_2$ sym. stretch	3227 (w)<br>3152 (w)	3457<br>3447	$–NH_2$ sym. stretch
2958 (vs)	3110	$–CH_3$ asym. stretch	2968 (vs)	3100<br>3032<br>2963	C–H asym. stretch from CH_3 (*t*Bu)<br>$–CH_2–$ asym. stretch (BAPP)
2930 (m)	3050	$–CH_2–$ asym. stretch (BAPP)<br>C–H sym. stretch from CH_3 (*t*Bu)	2934 (m)<br>2925 (m)		$–CH_2–$ sym. stretch (BAPP ring and tail)<br>$–CH_3$ sym. stretch
2859 (w)	2972,<br>2924	$–CH_2–$ sym. stretch (BAPP ring and tail)	2872 (m)<br>2820 (m)<br>2673 (vw)	2860<br>2821	$–CH_2–$ sym. stretch (BAPP ring and tail)
1584 (w)	1596	$–NH_2$ scissoring	1590 (w)	1601	$–NH_2$ scissoring

1466-1430	1432	–CH_2– scissoring	1460-1430 (m)	1423	–CH_2– scissoring<br>–CH_3 scissoring
1383 (m)<br>1361 (s)	1336	–CH_3 umbrella deformation (*t*Bu)<br>–CH_2– wagging (BAPP ring and tail)	1383 (m)<br>1361 (s)	1336	–CH_3 umbrella deformation (*t*Bu)<br>–CH_2– wagging (BAPP ring and tail)
			1347 (w)	1344	–CH_2– twisting
	1297	–CH_2– twisting	1310 (vw)		C–N stretch,<br>–CH_2– twisting
			1301 (w)<br>1270 (w)<br>1263 (w)	1291	C–N stretch,<br>–CH_2– twisting and wagging
1231 (s)<br>1210 (m)	1227<br>1198	–CH_3 wagging (deformation *t*Bu)	1239 (m)<br>1204 (m)	1250<br>1193	–CH_2– twisting (BAPP ring and ring)<br>–CH_3 wagging deformation of *t*Bu
1175 (s)<br>1137 (w)<br>1120 (w)	1151	C–N stretch	1185 (s)		–NH_2 twisting
			1146 (w)<br>1128 (w)		C–N stretch
-	-	-	1104 (w)		–NH_2 wagging
1091 (vw)<br>1083 (vw)	1083	Si–O stretch<br>C–C stretch	-	1092	Si–O stretch<br>C–C stretch
1036 (vs)	1038	Si–O–C asym. stretch<br>–CH_3 scissoring	1040 (vs)	1030	Si–O–C asym. stretch,<br>–CH_2– stocking (BAPP)<br>–NH_2 wagging
1012 (s)	1009	C–C twisting (*t*Bu)	1004 (vs)		–CH_3 twisting
998 (vs)<br>993 (vs)	-	Si-O-C asym. stretch	987 (vs)<br>961 (m)	965	C-C stretch BAPP ring
974 (m)<br>952 (m)	972	–NH_2 wagging	940 (w)	914	–NH_2 twisting
882 (w)	896	–CH_2– twisting (BAPP tail)		874	–NH_2 wagging
858 (vw)	836<br>808	Si–O stretch,<br>C–C stretch (*t*Bu)	842 (vw)	832	C-C stretch (*t*BuO)
813 (m)		Si–O stretch,<br>C–C stretch (*t*Bu)<br>–CH_2– twisting (BAPP tail)	820 (s)		–NH_2 scissoring
793 (m)			802 (s)	795	
734 (w)			770 (m)		
723 (vw)	711	Si–O–C stretch	728 (w)	723	Si–O–C stretch
688 (m)<br>677 (m)<br>657 (m)<br>607 (vw)	672	Si–S stretch,<br>deformation of *t*Bu	683 (s)<br>650 (s)	681<br>662<br>614	Si–S stretch,<br>deformation of *t*Bu
535 (m)	558	Si–S stretch	544 (s)	568	Si–S stretch

S2. Supporting figures

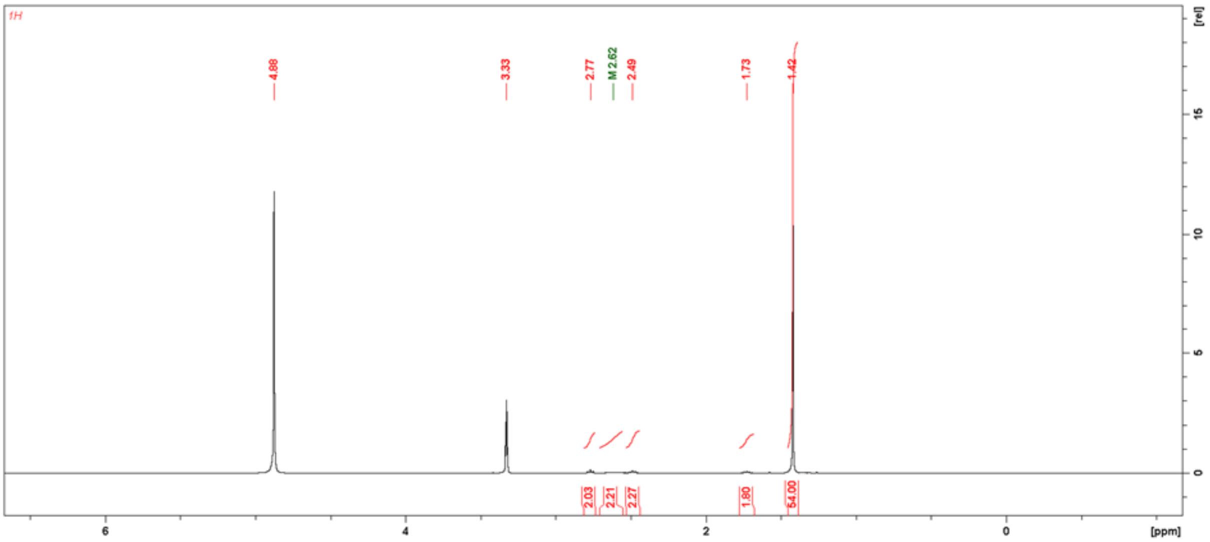

Figure S1 ^{1}H-NMR spectrum of **1** crystals dissolved in CH_3OH-d_4 with integration.

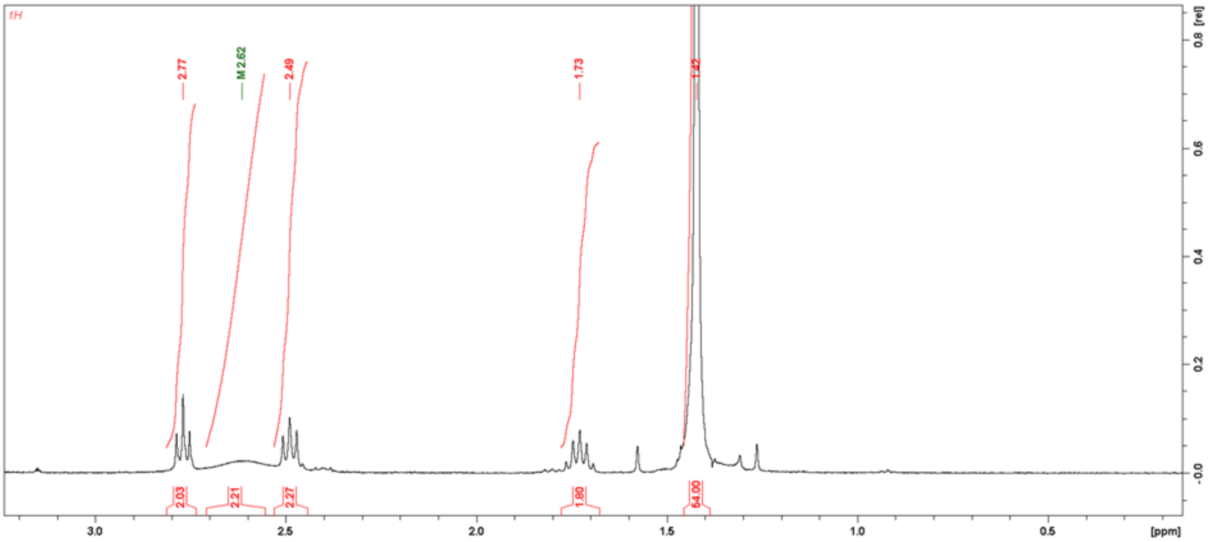

Figure S2 ^{1}H-NMR spectrum of **1** crystals dissolved in CH_3OH-d_4 with integration in the range from 0 ppm to 3 ppm.

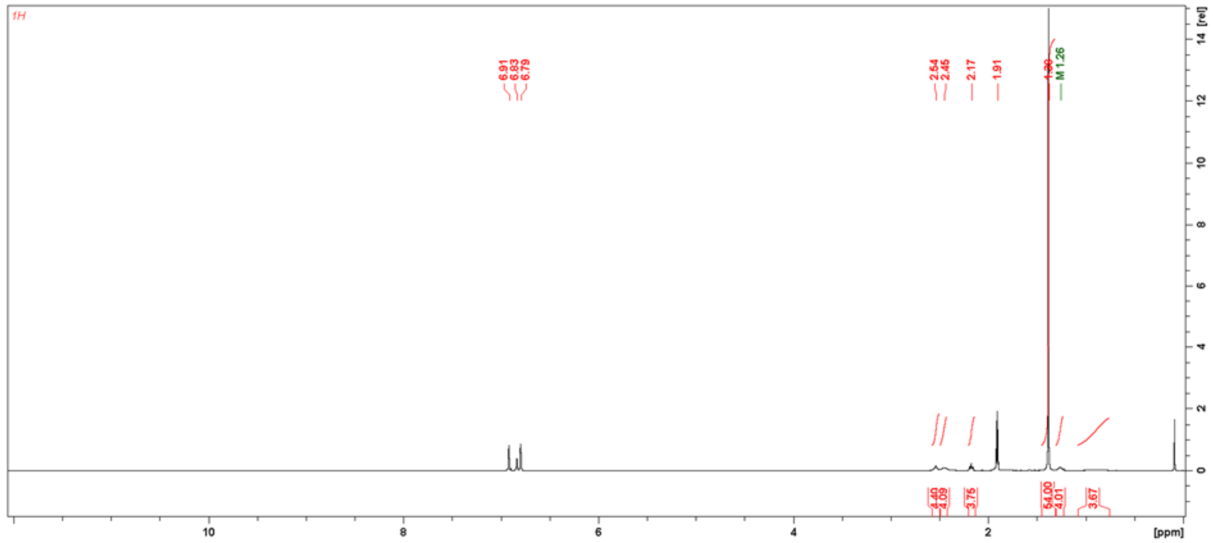

Figure S3 ^{1}H-NMR spectrum of **2** crystals dissolved in toluene-d$_6$ with integration.

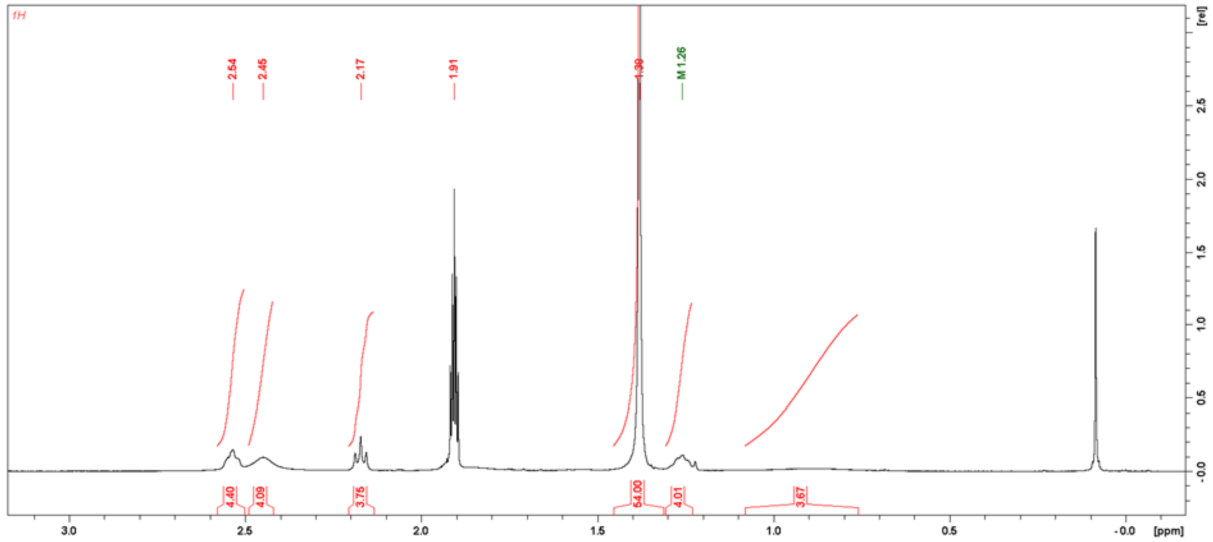

Figure S4 ^{1}H-NMR spectrum of **2** crystals dissolved in toluene-d$_6$ with integration in the range from 0 ppm to 3 ppm.

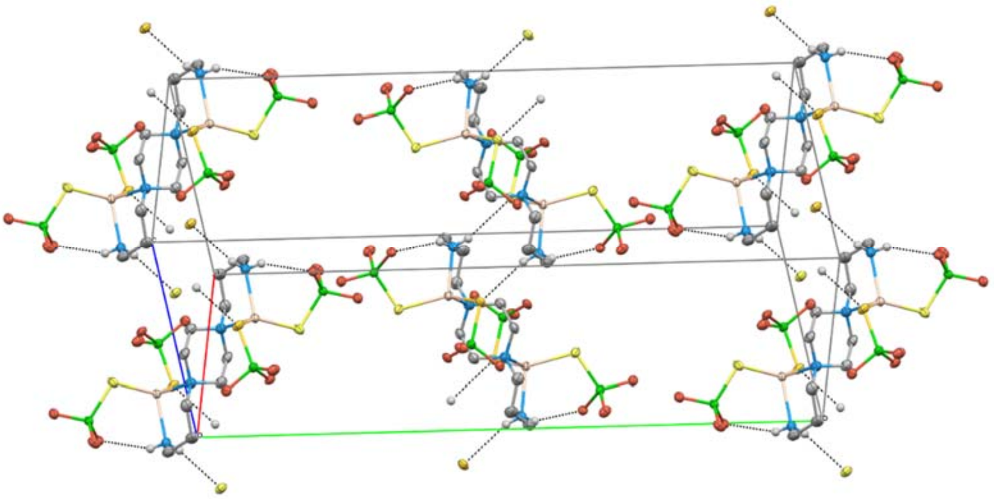

Figure S5 Partial packing view of **1**, showing the intermolecular interactions. Dashed lines indicate hydrogen bonds.

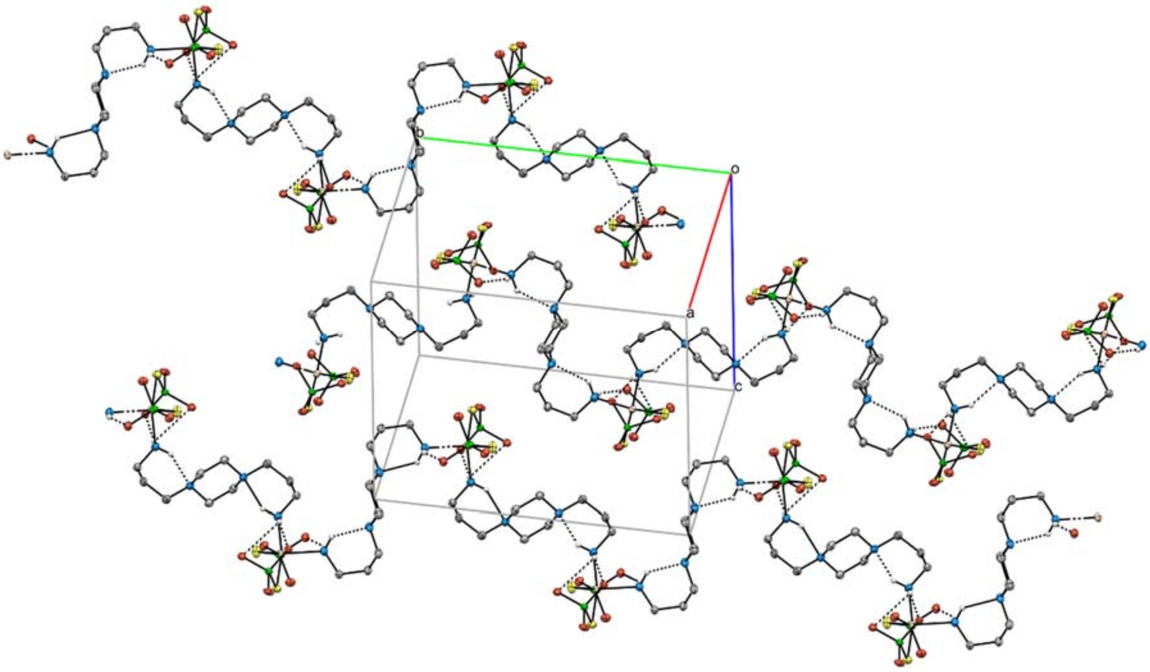

Figure S6 Partial packing view of **2**, showing the intermolecular interactions. Dashed lines indicate hydrogen bonds.

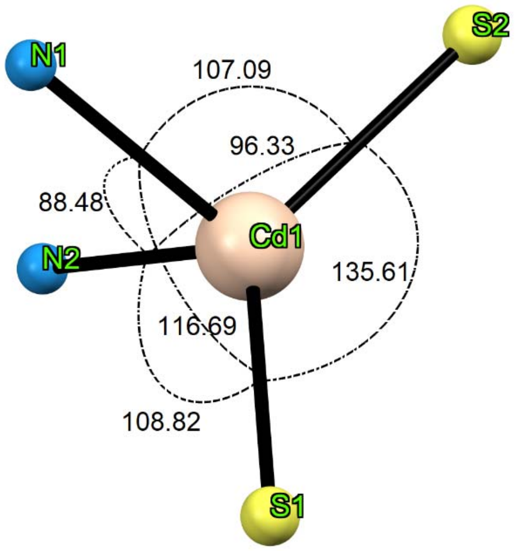

Figure S7 Geometry of Cd centre in compound **1** with angles and their values indicated.

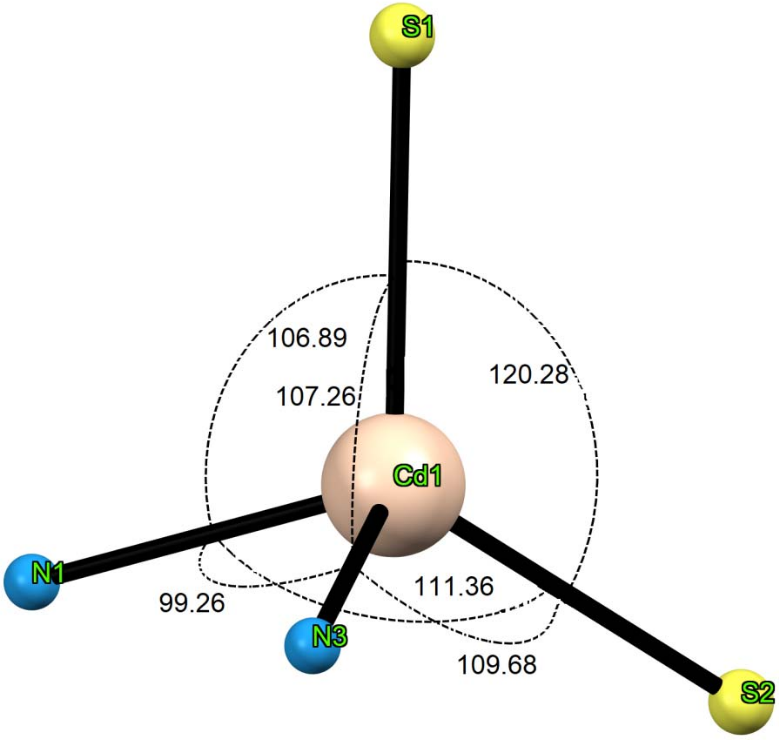

Figure S8 Geometry of Cd centre in compound **2** with angles and their values indicated.

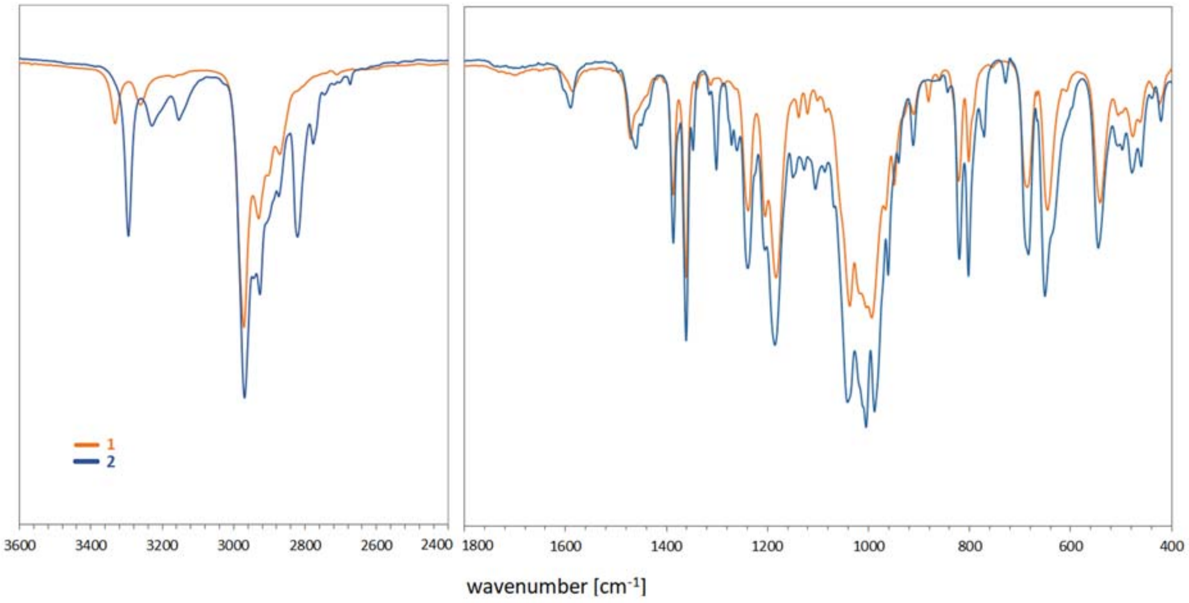

Figure S9 Experimental FT-IR spectra of complexes **1** (orange line) and **2** (blue line).

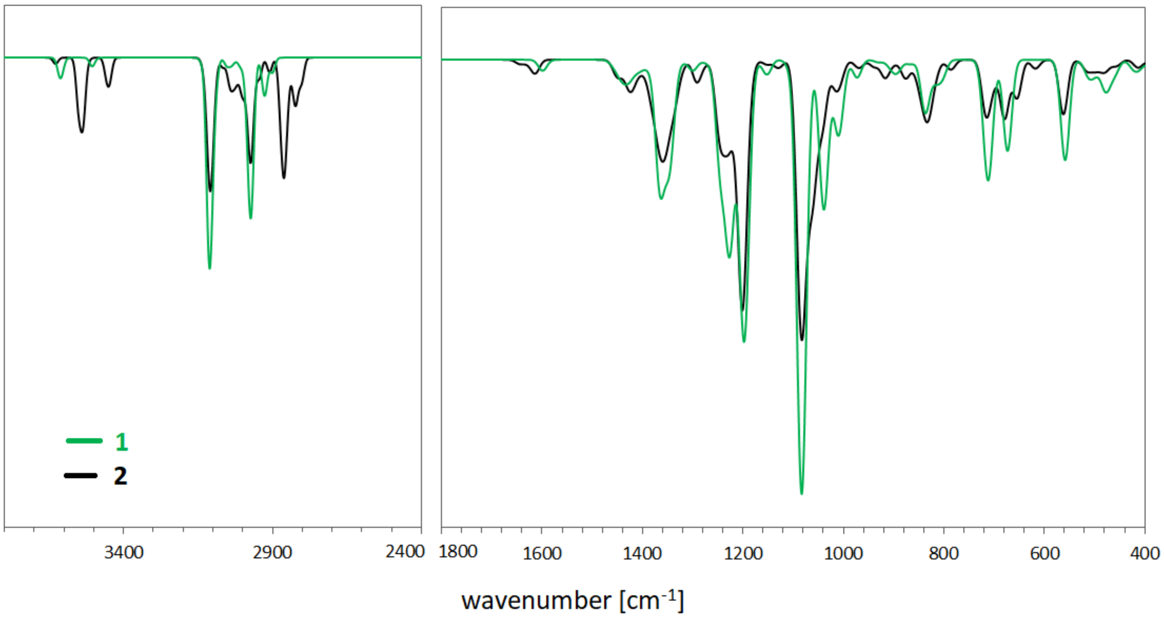

Figure S10 Calculated FT-IR spectra of complexes **1** (green line) and **2** (black line).

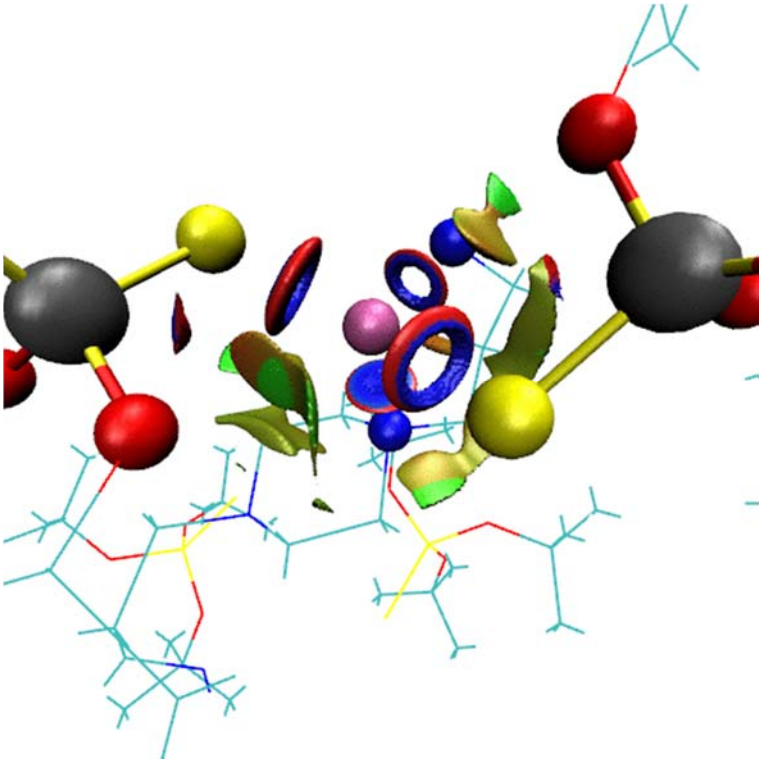

Figure S11 Three-dimensional visualizations of the coordination centers in **1**.

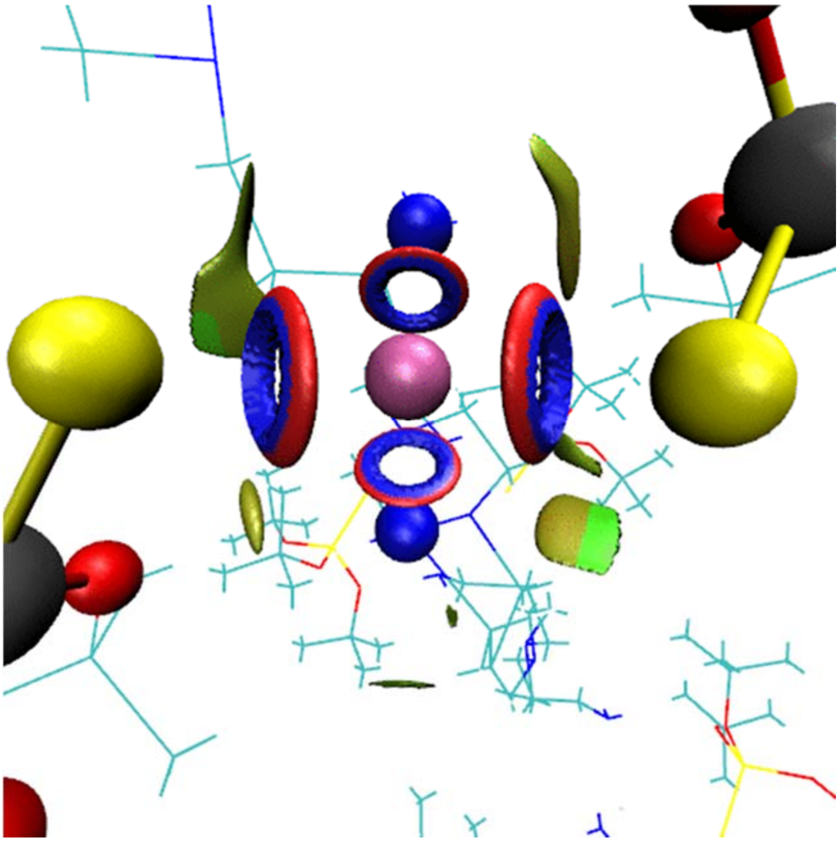

Figure S12 Three-dimensional visualizations of the coordination centers in **2**.

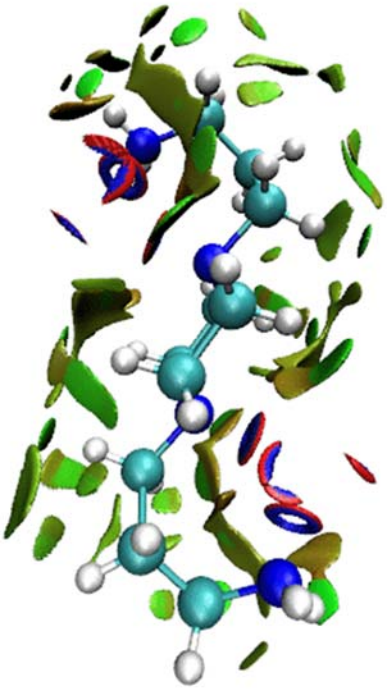

Figure S13 Three-dimensional visualizations of the coordination centers in **1**.

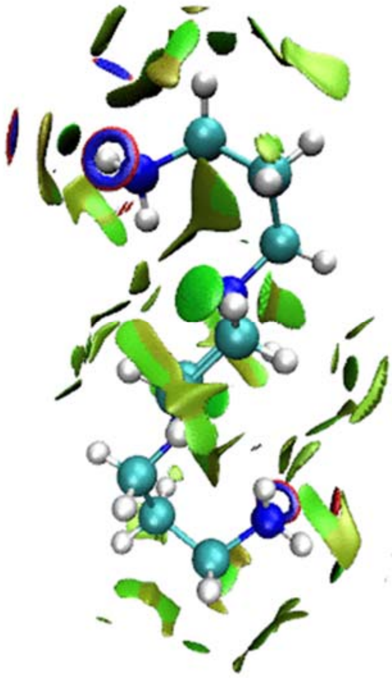

Figure S14 Three-dimensional visualizations of the coordination centers in **2**.

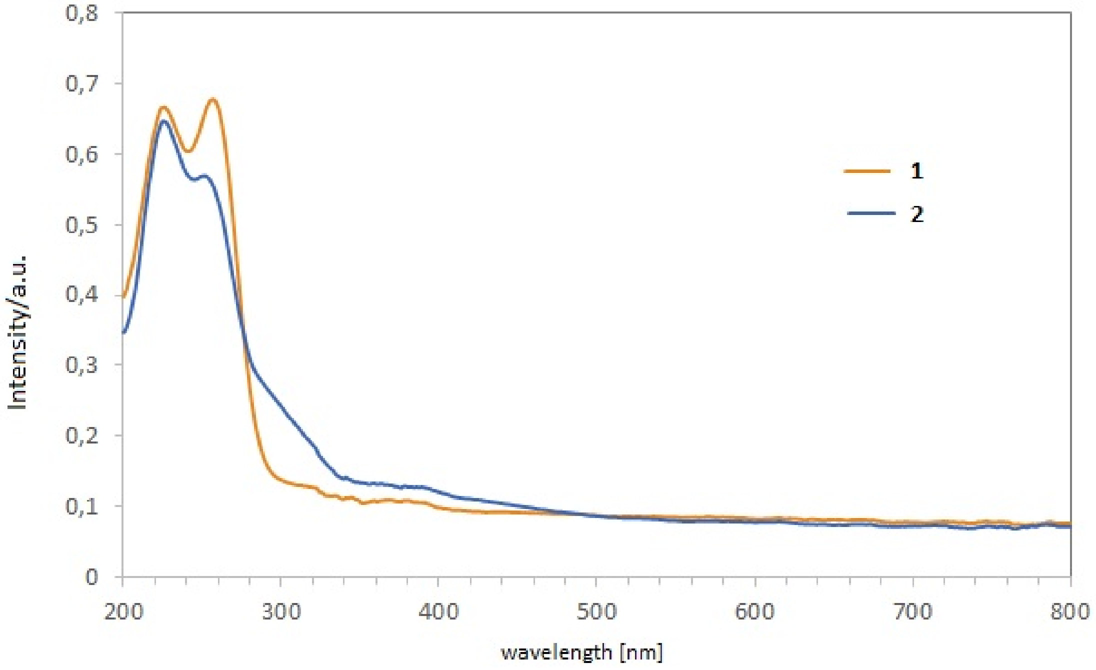

Figure S15 UV–vis absorption spectra of **1** and **2**.

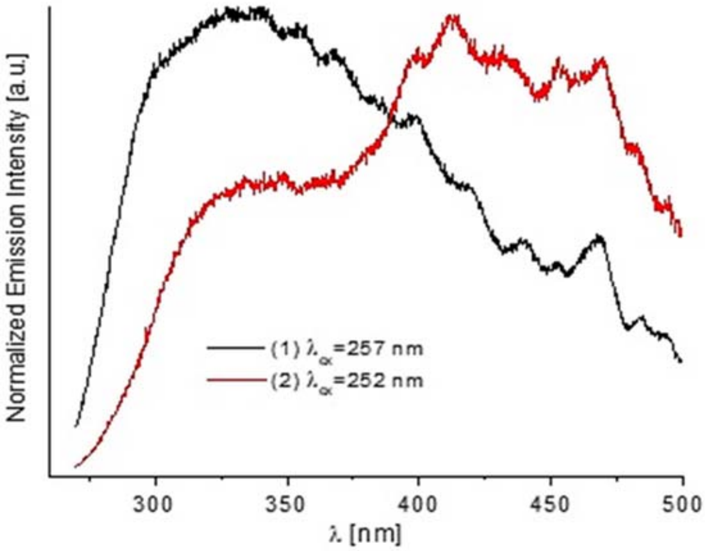

Figure S16 Emission spectra of **1** and **2** recorded at the wavelength corresponding to the maximum absorption of these systems.
